# Conversion of phosphoenolpyruvate to pyruvate in *Thermoanaerobacterium saccharolyticum*

**DOI:** 10.1016/j.mec.2020.e00122

**Published:** 2020-01-23

**Authors:** Jingxuan Cui, Marybeth I. Maloney, Daniel G. Olson, Lee R. Lynd

**Affiliations:** a Department of Biological Sciences, Dartmouth College, Hanover, NH, 03755, USA; b Center for Bioenergy Innovation, Oak Ridge National Laboratory, Oak Ridge, TN, 37830, USA; c Thayer School of Engineering, Dartmouth College, Hanover, NH, 03755, USA

**Keywords:** Pyruvate kinase, Pyruvate phosphate dikinase, Pyruvate, Ethanol, *Thermoanaerobacterium saccharolyticum*, Consolidated bioprocessing

## Abstract

*Thermoanaerobacterium saccharolyticum* is an anaerobic thermophile that can ferment hemicellulose to produce biofuels, such as ethanol. It has been engineered to produce ethanol at high yield and titer. *T. saccharolyticum* uses the Embden-Meyerhof-Parnas (EMP) pathway for glycolysis. However, the genes and enzymes used in each step of the EMP pathway in *T. saccharolyticum* are not completely known. In *T. saccharolyticum*, both pyruvate kinase (PYK) and pyruvate phosphate dikinase (PPDK) are highly expressed based on transcriptomic and proteomic data. Both enzymes catalyze the formation of pyruvate from phosphoenolpyruvate (PEP). PYK is typically the last step of EMP glycolysis pathway while PPDK is reversible and is found mostly in C4 plants and some microorganisms. It is not clear what role PYK and PPDK play in *T. saccharolyticum* metabolism and fermentation pathways and whether both are necessary. In this study we deleted the *ppdk* gene in wild type and homoethanologen strains of *T. saccharolyticum* and showed that it is not essential for growth or ethanol production.

## Highlights

• Deleted gene encoding *ppdk* in two strains of *Thermoanaerobacterium saccharolyticum.*
• Deletion of *ppdk* does not affect growth, pyruvate kinase activity, or fermentation.
• Pyruvate kinase appears to be essential in *Thermoanaerobacterium saccharolyticum*.

## 1 Introduction

Lignocellulosic biomass is a sustainable feedstock for biofuels, however it is not currently commercially viable due to its recalcitrance (Wyman, 2007). Consolidated bioprocessing (CBP) is a leading strategy for low-cost conversion of lignocellulosic biomass into fuels, wherein biomass solubilization and fermentation of released sugars are combined in one step (Olson et al., 2012). *Thermoanaerobacterium saccharolyticum* is a thermophilic anaerobe that has the capability to solubilize hemicellulose components of plant biomass and produce ethanol as a fermentation production, and is thus a candidate for CBP (Olson et al., 2012). Wild type *T. saccharolyticum* produces acetic acid, lactic acid, CO_2_ and H_2_ together with ethanol. Deleting these competing pathways successfully increased ethanol production from *T. saccharolyticum,* reaching a yield of 90% of the theoretical maximum and titer of 70 ​g/L (Shaw et al., 2008; Herring et al., 2016). The ability to produce ethanol at high yield and titer provides an example for metabolic engineering of other thermophilic bacteria. Furthermore, it would be useful to be able to transfer this ethanol production ability to other organisms. To do this, we need a better understanding of the genes and enzymes responsible for all of the steps in the cellobiose to ethanol pathway.

**Fig. 1:**
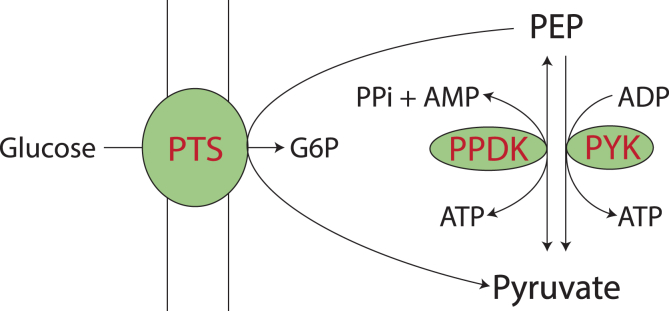
**Possible routes for pyruvate production from PEP in T. saccharolyticum.** Metabolites are shown in black and enzymes involved are shown red. Abbreviations used are G6P: glucose 6-phosphate; PEP: phosphoenolpyruvate; PTS: phosphoenolpyruvate-dependent sugar phosphotransferase system; PPDK: pyruvate phosphate dikinase; PYK: pyruvate kinase.

The enzymes (and their corresponding genes) responsible for ethanol production downstream of glycolysis have been well studied in *T. saccharolyticum*. Briefly, the combination of pyruvate ferredoxin oxidoreductase (PFOR), ferredoxin:NAD oxidoreductase (FNOR), aldehyde dehydrogenase (ALDH) and alcohol dehydrogenase (ADH) catalyze an electron balanced pathway to produce ethanol from the pyruvate and NADH generated in glycolysis (Zhou et al., 2015; Lo et al., 2015; Zheng et al., 2017a). However, the enzymes responsible for glycolysis, especially for pyruvate production have not been studied in *T. saccharolyticum.* Based on the current genome annotation (Genbank CP003184.1), there are three different routes for pyruvate production from phosphoenolpyruvate (PEP) in *T. saccharolyticum:* pyruvate kinase (PYK, E.C. 2.7.1.40, encoded by Tsac_1363), pyruvate phosphate dikinase (PPDK, EC 2.7.9.1, encoded by Tsac_2038) and the phosphoenolpyruvate-dependent sugar phosphotransferase system (PTS) (Fig. 1). There are multiple PTS gene clusters in *T. saccharolyticum* with specificity for different sugars, including glucose, fructose, cellobiose and xylose. Transcriptomic and proteomic data suggests that the expression levels of different PTS gene clusters change in response to varying substrates (Currie et al., 2015), suggesting that the PTS system is highly regulated. The stoichiometry for PTS system is PEP ​+ ​glucose → pyruvate ​+ ​G6P. Since one PEP is converted to pyruvate per glucose transport event, but glucose is converted to two PEP molecules by glycolysis, the PTS system can account for at most half of the PEP → pyruvate flux (assuming negligible carbon leaves at intermediate stages of glycolysis). Both PYK and PPDK are expressed at high levels in *T. saccharolyticum*, independent of substrate*,* based on transcriptomic and proteomic analysis (Currie et al., 2014), suggesting the possibility that one or both enzymes play a significant role in *T. saccharolyticum* metabolism.

In the canonical glycolysis pathway, the last step is catalyzed by PYK, which irreversibly converts PEP and ADP to pyruvate and ATP. PPDK is found in plants and a variety of microorganisms, catalyzing the reversible conversion of PEP, AMP and PPi to pyruvate, ATP and Pi. In organisms where PYK is absent, such as *Entamoeba histolytica, Clostridium symbiosum* and *Clostridium thermocellum*, PPDK functions as the substitutes for PYK in the direction of ATP synthesis (Pineda et al., 2015; McGuire et al., 1998; Olson et al., 2017; Saavedra et al., 2005); while in other organisms, such as *Acetobacter xylinum* (Benziman and Eizen, 1971)*,* the photosynthetic bacterium *Rhodospirillum rubrum* (Ernst et al., 1986) and plants (Lappe et al., 2018), PPDK functions in gluconeogenesis and is responsible for PEP production. Genes encoding typical gluconeogenic enzymes such as PEP synthase and fructose 1,6-biosphosphatase are absent in *T. saccharolyticum* genome, and there are no known conditions under which gluconeogenesis occurs, thus PPDK likely participate in catabolism (glycolysis) instead of gluconeogenesis.

Our aim for this project is to answer the question of whether PYK and PPDK are both required in the glycolysis of *T. saccharolyticum* and their role in strains engineered for high-titer ethanol production. To answer these questions, we attempted to delete the genes encoding PYK and PPDK in both wild type and homoethanologen (Herring et al., 2016) (i.e. engineered to produce only ethanol as a fermentation product) strains of *T. saccharolyticum*.

## 2 Materials and Methods

### 2.1 Media and growth conditions

All reagents used in this study were obtained from Sigma Aldrich or Fisher Scientific, unless otherwise stated.

*T. saccharolyticum* was grown at 55 ​°C under anaerobic conditions in conical tubes in an anaerobic chamber (Coy Laboratory Products, MI, USA). Complex CTFUD medium was prepared as previously described (Cui et al., 2019), and used to culture *T. saccharolyticum* in preparation for transformation, or to harvest genomic DNA for whole-genome sequencing. 5 ​g/L cellobiose was used as substrate. For regular culture and FUDR selection, the pH of the medium was adjusted to 6.0, and for kanamycin selection, the pH was adjusted to 6.7.

The defined MTC-6 medium was prepared as previously described (Cui et al., 2019) and used for batch fermentations, and to prepare cell pellets for enzyme assays. Cellobiose concentrations of 5 ​g/L and 20 ​g/L were used for fermentation end-product analysis.

To measure specific growth rates, cells were grown in 200 ​μL of MTC-6 medium containing 5 ​g/L cellobiose in a 96-well plate. Growth was measured by monitoring absorbance at 600 ​nm every 5 ​min for 72 ​h in a BioTek Powerwave XS plate reader as previously described (Olson and Lynd, 2012).

### 2.2 Strain and plasmid construction

A complete list of strains and plasmids is presented in Table 1. The plasmid design is based on plasmid pLT-26 as previously described (Tian et al., 2016a). Each plasmid contains five segments, a replication origin for *E. coli*, a 5′ homology region upstream of the coding sequence (CDS, i.e. start to stop codon, inclusive) of the gene of interest, a 3’ homology region downstream of the gene of interest, the selection marker cassette, and an internal homology region within the gene of interest (supplementary Figure 1). All fragments were generated by PCR or ordered as gBlocks® Gene Fragments from Integrated DNA Technology (IDT, CA, USA), and assembled together to form the circular plasmid by isothermal assembly using NEBuilder® HiFi DNA Assembly Master Mix (New England Biolabs, MA, USA). The assembled plasmids were transformed into *E. coli* DH5α chemically competent cells from New England Biolabs. DNA purification of the fragments was performed using a commercial kit from Zymo Research, and plasmid extraction was performed using the Qiagen miniprep kit. All plasmids were confirmed by Sanger Sequencing (Genewiz, NJ, USA). Strains were constructed by the markerless replacement system as described previously (Cui et al., 2019).

**Table 1:** Plasmids and strains used in this study.

Strains or plasmids	Description	GenBank accession number	Reference
**pJC12**	plasmid to delete the entire CDS of Tsac*_2038* (*ppdk)* gene	MN544098	This study
**pJC13**	plasmid to delete the entire CDS of Tsac_1363 (*PYK*) gene	MN544099	This study
**pJC15**	plasmid to delete 84% of the CDS of Tsac_1363 (PYK) gene	MN544100	This study
**pJC16**	plasmid to delete 71% of the CDS of Tsac_1363 (PYK) gene	MN544101	This study
**pJC17**	plasmid to delete 49% of the CDS of Tsac_1363 (PYK) gene	MN544102	This study
**LL1305**	LL1025 Δ*tdk* (wild type)	SRP144560	Tian et al. (2016b)
**LL1328**	LL1049 Δ*tdk* (homoethanologen)	SRP144558	Zheng et al. (2017b)
**LL1580**	LL1305 Δ*ppdk*	SRP164859	This study
**LL1609**	LL1305 Δ*pyk* (34% deletion)	SRP164878	This study
**LL1610**	LL1580 Δ*pyk* (52% deletion)	SRP164876	This study
**LL1686**	LL1328 Δ*ppdk*	SRP237536	This study

### 2.3 Fermentation end products analysis

*T. saccharolyticum* cells were cultured for 72 ​h in MTC-6 medium containing 5 ​g/L or 20 ​g/L initial cellobiose in 15 ​mL conical centrifuge tubes (Westnet MA, USA), kept at 55 ​°C in an anaerobic chamber (10% CO_2_, 1–5% H_2_, <5 ​ppm O_2_ with N_2_ comprising the remainder).

At the end of the fermentation, 1 ​mL of culture was collected from each tube and centrifuged at 21,100 relative centrifugal force (RCF) for 5 ​min. Supernatant was collected and the fermentation products were quantified by high pressure liquid chromatography (HPLC) as previously described (Holwerda et al., 2014).

Ethanol yield was calculated as the percentage of theoretical yield based on the amount of ethanol produced and substrate consumed:

(3) $$Ethanol\;yield\left(percent\;theoretical\right)=\frac{Produced\;Ethanol\;\left(mM\right)}{Consumed\;Cellobiose\left(mM\right)*4}*100\%$$

### 2.4 Enzyme assays

#### 2.4.1 Preparation of cell-free extracts

*T. saccharolyticum* cells were grown in an anaerobic chamber in MTC-6 medium containing 5 ​g/L cellobiose and harvested in the exponential phase of growth (OD_600_ between 0.6 and 0.8). To prepare cell-free extracts, cells were collected by centrifugation at 7,000 RCF for 15 ​min and washed under anaerobic conditions with 1 ​mL of a buffer that contains 100 ​mM Tris-HCl (pH 7.5) and 0.5 ​mM dithiothreitol (DTT). Cells from a 50 ​mL culture were resuspended in 1 ​mL of the washing buffer. Resuspended cells were lysed by adding 1 ​μL of Ready-Lyse lysozyme solution (Epicentre, WI, USA) and incubated at room temperature (22–27 ​°C) for 20 ​min. Then 1 ​μL of DNase I solution (Thermo Scientific, MA, USA) was added to reduce the viscosity of the solution and it was allowed to incubate for an additional 10 ​min. The crude lysate was centrifuged at 21,100 RCF for 5 ​min in the anaerobic chamber and the supernatant was collected as cell-free extract (CFE). The total amount of protein in the CFE was determined by Bradford assay, using bovine serum albumin (Thermo Scientific) as the standard.

#### 2.4.2 General assay conditions

All enzyme assays were performed in an anaerobic chamber at 55 ​°C and pH 7.5. Enzyme activities were assayed in a reduced-volume quartz cuvette (part number 29MES10; Precision Cells Inc., NY, USA) with a 1.0 ​cm path length at a final volume of 1 ​mL using an Agilent Technologies (Santa Clara, CA, USA) 8453 UV–visible spectrophotometer with water-bath heating set at 55 ​°C, as previously described (Olson et al., 2017). The units for all enzyme activities are expressed as μmol of product/min/mg of cell extract protein. For each enzyme assay, at least two concentrations of cell extract were used to confirm that specific activity was proportional to the amount of extract added. All chemicals and coupling enzymes were purchased from Sigma. For assays involving NADH or NADPH, an extinction coefficient of 6.3 ​mM^-1^ ​cm^-1^ was used.

#### 2.4.3 Alcohol dehydrogenase (ADH, EC 1.1.1.1 & EC 1.1.1.2)

ADH was measured in the direction of acetaldehyde reduction. The assay mixture contained 50 ​mM Tris-HCl buffer (pH 7.5), 0.3 ​mM NAD(P)H, 5 ​mM MgCl_2_, 10 ​mM acetaldehyde, 0.5 ​mM DTT, and cell-free extract. The reaction was started by adding acetaldehyde, and progress was followed by monitoring the decrease in absorbance at 340 ​nm.

#### 2.4.4 Pyruvate kinase (PYK, EC 2.7.1.40)

PYK was measured by coupled assay with lactate dehydrogenase (LDH). The assay buffer contained 50 ​mM Tris-HCl, pH 7.5 (at 55 ​°C), 5 ​mM dithiothreitol (DTT), 10 ​mM KCl, 12 ​mM MgCl_2_, 10 ​mM ADP, 0.1 ​mM 3-phosphoglyceric acid (3PG), 5 ​mM PEP, 12 U LDH enzyme (Sigma L2500) and 0.3 ​mM NADH. Water was added to a final volume of 1.0 ​mL. PEP was converted to pyruvate, which was converted to lactate by LDH with the concomitant reduction of NADH to NAD^+^. The decrease in NADH concentration over time was measured at 340 ​nm by spectrophotometer. The reaction could be started with either ADP or PEP. For routine assays it was started with PEP. The assay is sensitive to MgCl_2_ levels and did not work when MgCl_2_ was lower than 2 ​mM.

#### 2.4.5 Pyruvate phosphate dikinase activity (PPDK, EC 2.7.9.1)

PPDK was measured by coupled assay with LDH. The assay buffer contained 50 ​mM Tris-HCl, pH 7.5 (at 55 ​°C), 5 ​mM DTT, 0.3 ​mM NADH, 5 ​mM MgCl_2_, 20 ​mM NH_4_Cl, 2 ​mM PEP, 12 U LDH enzyme (Sigma L2500), 2 ​mM AMP and 1 ​mM pyrophosphate (PPi). The reaction was started with the addition of PP_i_, although it could also be started by adding AMP or PEP. The decrease in NADH concentration over time was measured at 340 ​nm by spectrophotometer. NH_4_Cl is an activator for PPDK activity and is essential to the enzyme assay. At 55 ​°C, the mixture of 5 ​mM MgCl_2_ and 1 ​mM PP_i_ forms a precipitate after 1 ​h, so the assay must be done within 1 ​h for accurate measurement. Slight increases in the concentration of either compound resulted in rapid precipitation.

### 2.5 Whole genome resequencing

Whole genome resequencing for all strains constructed in this study was used to verify strain construction and check for secondary mutations, as described before (Zhou et al., 2015). Genomic DNA was prepared and submitted to the Joint Genome Institute (JGI) for sequencing with an Illumina MiSeq instrument. Raw data is available from the JGI Sequence Read Archive (accession numbers are presented in Table 1). Data was analyzed with the CLC Genomic Workbench version 11.0.1, (Qiagen Inc., Hilden, Germany), as previously described (Zhou et al., 2015).

## 3 Results and discussion

### 3.1 Enzyme activity of PYK and PPDK in *T. saccharolyticum*

**Fig. 2:**
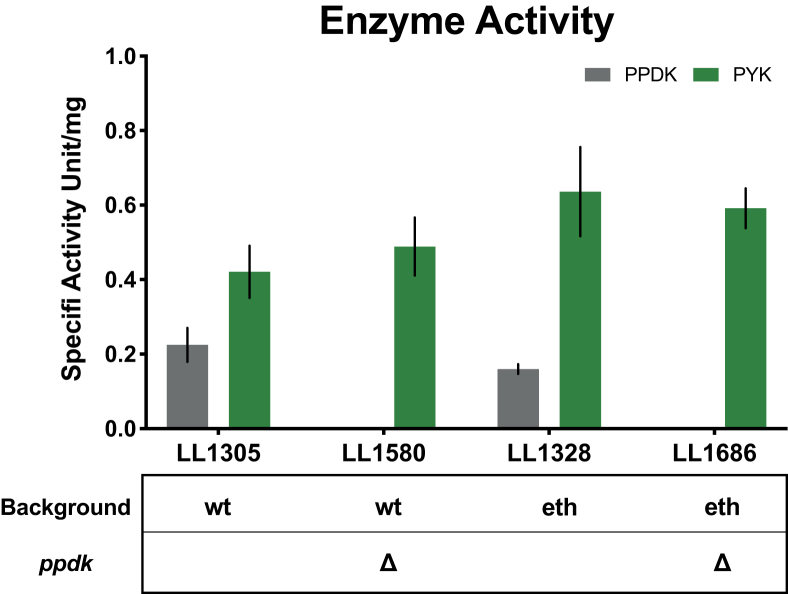
**Enzyme activities of T. saccharolyticum strains.** Pyruvate kinase (PYK, shown in green) and pyruvate phosphate dikinase (PPDK, shown in grey) were measured in wild type strain (LL1305), homoethanologen strain (LL1328) and the Δppdk strains (LL1580 and LL1686). wt stands for wild type, eth stands for homoethanologen. All strains were grown in MTC-6 medium containing 5 ​g/L initial cellobiose and collected at mid-log phase of growth. Error bars represent one standard deviation (n ​= ​6).

To confirm that the PPDK and PYK enzymes are functionally active in cells, we tested the PYK and PPDK activity in strains with both wild type (LL1305) and homoethanologen (LL1328) levels of ethanol production. We were able to detect both enzyme activities in the cell-free extract of LL1305 and LL1328 (Fig. 2). The PYK activity was higher than PPDK activity in both strains. The PYK activity was almost 2-fold higher than PPDK in LL1305 (wild type) and more than 3-fold higher in LL1328 (homoethanologen). Comparing LL1328 to LL1305, the PYK activity was about 1.5-fold higher while PPDK was much lower.

In organisms where both PPDK and PYK are present, and PPDK plays an important role in metabolism, the specific activity of PPDK detected from cell-free extract is always several fold higher than PYK. For example, in *Entamoeoba histolytica*, although both *ppdk* and *pyk* encoding genes are present on the genome, the PPDK activity is 0.34 +∖- 0.12, which is 10 fold higher than PYK (0.032±0.016) (Pineda et al., 2015). In *Caldicellulosiruptor saccharolyticus*, the PPDK activity (0.42±0.09) is 2 fold higher than PYK (0.18±0.05) (Bielen et al., 2010).

### 3.2 Deletion of the *ppdk* gene

To determine the role of PPDK in metabolism, we deleted the entire coding sequence of the *ppdk* gene in both LL1305 (wild type) and LL1328 (homoethanologen) strains, resulting in strains LL1580 and LL1686 respectively. Deletion of the *ppdk* gene was confirmed by PCR and whole genome resequencing (see Materials and Methods).

To investigate whether PPDK is essential for *T. saccharolyticum* metabolism, we first compared the growth of Δ*ppdk* strains. In the wild type background, *ppdk* deletion doesn’t affect growth on cellobiose, supported by the same growth rate of strain LL1580 compared to its parent strain LL1305 (Table 2). However, in the homoethanologen background (LL1686 vs. LL1328), deleting *ppdk* gene decreased the growth rate by about 30% (Table 2), suggesting that PPDK is more important in the homoethanologen strain.

**Table 2:** Maximum growth rate of Δppdk strains.

Strain	Genotype	Growth rate (1/h)
**LL1305**	Wild type (wt)	0.41 (0.03)^a^
**LL1328**	Homoethanologen (eth)	0.43 (0.02)
**LL1580**	wt Δ*ppdk*	0.42 (0.04)
**LL1686**	eth Δ*ppdk*	0.27 (0.01)

a Numbers indicate average growth rate from three replicates and numbers in parentheses represent the standard deviation.

Enzyme activities of PYK and PPDK were measured in strain LL1580 and LL1686 and compared to the parent strains (Fig. 2). In both deletion strains, the PPDK activity was entirely eliminated, confirming the deletion of the *ppdk* gene. No significant changes in PYK activity were observed as a result of the *ppdk* deletions. These data suggest that PYK alone is enough to support growth in *T. saccharolyticum* and is likely the main enzyme used for pyruvate generation from glycolysis.

The removal of PP_i_ (either by hydrolysis to P_i_ or reincorporation into ATP) is an indispensable reaction in metabolism since PP_i_ is a byproduct of various biosynthetic reactions. These reactions, such as DNA and RNA biosynthesis are often close to equilibrium and effective removal of PP_i_ drives them forward (Chen et al., 1990). Many microbes have soluble pyrophosphatase enzymes to limit PP_i_ accumulation. In *T. saccharolyticum*, there is no cytosolic pyrophosphatase annotated in the genome, however a membrane bound pyrophosphatase that couples the PP_i_ hydrolysis with H^+^ pumping (Tsac_2336) might be used instead. Tsac_2336 is expressed at a relatively high level but the protein abundance is much lower than PPDK (Currie et al., 2015). Thus, one possible physiological function of PPDK is consumption of PP_i_ to prevent inhibition of biosynthetic reactions.

**Fig. 3:**
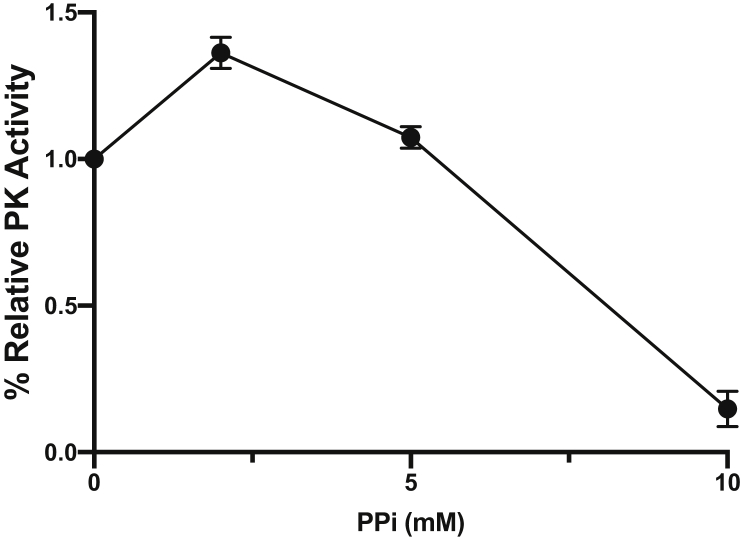
**Influence of PPi concentration on pyruvate kinase activity.** PYK activity was measured in the cell-free extract of the homoethanologen strain of *T. saccharolyticum* (LL1328) with addition of 2 ​mM, 5 ​mM or 10 ​mM PP_i_. Relative activity was calculated based on the no-inhibitor condition. Error bars represent one standard deviation (n ​= ​2).

It has been reported that in *C. saccharolyticus* and *Trypanosoma cruzi* the PYK is inhibited by PP_i_, thus the concentration of PP_i_ regulates whether PYK or PPDK is used for glycolysis (Bielen et al., 2010; Acosta et al., 2004). To test whether the same regulation exists in *T. saccharolyticum,* we tested the influence of different concentrations of PPi on pyruvate kinase activity (Fig. 3). We only found PPi inhibition on PYK at very high concentration, with up to 5 ​mM PP_i_, PYK was still 100% active and only at 10 ​mM PPi was inhibition observed. On the other hand, in *C. saccharolyticus*, PYK activity is inhibited by PP_i_ concentrations as low as 1 mM. Although we have not measured the PP_i_ concentration in *T. saccharolyticum,* the high flux through PYK in Δ*ppdk* strains suggests that the intracellular PP_i_ concentration is below the inhibition threshold. For comparison, the intracellular PP_i_ concentration in *E. histolytica* is 0.45 ​± ​0.1 ​mM (Saavedra et al., 2007), for *E. coli* it is between 0.5 to 1 ​mM (Chen et al., 1990; Biochem and Kukko, 1982), and for *Anacystis nidutans* it is between 1-2 ​mM (Bornefeld, 1981).

### 3.3 Fermentation results from Δ*ppdk* strains

**Fig. 4:**
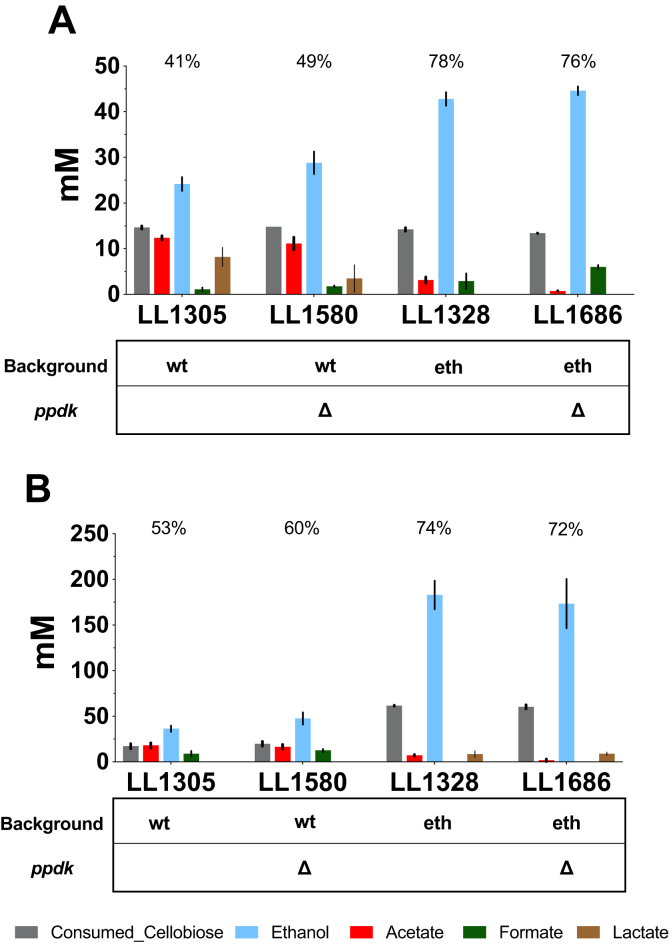
**Fermentation result of T. saccharolyticum ppdk deletion strains**. Data was collected from 72-h batch fermentations in MTC-6 medium containing 5 ​g/L (A) or 20 ​g/L (B) initial cellobiose. The number above the ethanol bar indicates ethanol yield, as a percentage of the theoretical maximum, which is 4 ​mol of ethanol per mole of cellobiose. wt stands for wild type background, eth stands for homoethanologen background. Error bars represent one standard deviation from three replicates.

Although deleting *ppdk* does not influence the growth of *T. saccharolyticum,* it’s possible that it will influence other metabolic pathways. So next we tested whether deleting the *ppdk* gene changes fermentation results of wild type and homoethanologen strains of *T. saccharolyticum.* The *ppdk* deletion strains, LL1580 and LL1686 and their parent strains LL1305 and LL1328 were prepared for batch fermentation using MTC-6 medium at two different substrate concentrations, 5 ​g/L and 20 ​g/L. Fermentation end products were analyzed after 72 ​h (Fig. 4).

In the wild type background (LL1580 vs. LL1305), deleting *ppdk* resulted in a slight but significant increase (p ​= ​0.04) in ethanol production from 5 ​g/L cellobiose, at the expense of other products such as acetate and lactate. The ethanol yield was increased by 20%. When grown on 20 ​g/L, the increase in ethanol yield was 13% and the difference was not significant from the wild type (p ​= ​0.06). In the homoethanologen background (LL1328 vs. LL1686), deleting *ppdk* did not change ethanol production.

### 3.4 Secondary mutations in Δ*ppdk* strains

Strains LL1580 and LL1686 were submitted for whole genome sequencing to analyze secondary mutations associated with the *ppdk* deletion. In LL1580, mutations in the DNA sequence that caused alterations in amino acid sequences were found in two genes, including the bifunctional aldehyde and alcohol dehydrogenase AdhE, and the flagellar motor switch protein FliN (Table 3). Another mutation was found at the promotor region of a RpiR family transcription regulator. In LL1686, 20 different sequence variations were found in one gene, Tsac_2318, which encodes a Uma2 family endonuclease (Table 3).

**Table 3:** Secondary mutations in T. saccharolyticum ppdk deletion strains.

Strain	Gene	Product	Type^c^	CDS change^d^	AA change	Frequency^e^
LL1580	Tsac_0416	AdhE^a^	SNV	1790C ​> ​A	T597K	100%
LL1580	Tsac_1793	FliN^b^	SNV	1065C ​> ​A	F355L	100%
LL1580	10 bp upstream of Tsac_1881	transcriptional regulator, RpiR family	SNV	C ​> ​T		100%
LL1686	Tsac_2318	Uma2 family endonuclease	Ins	153_154insG	F52fs	41%
			MNV	157_158delGCinsTT	A53F	41%
			SNV	161A ​> ​G	N54S	41%
			Ins	162_163insA	Y55fs	41%
			Del	365delG	R122fs	45%
			Ins	367_368insA	V123fs	45%
			SNV	394A ​> ​T	N132Y	39%
			SNV	399A ​> ​T		37%
			SNV	402A ​> ​G		37%
			SNV	405G ​> ​A		37%
			SNV	408G ​> ​A		37%
			SNV	411T ​> ​C		37%
			SNV	418A ​> ​G	I140V	37%
			SNV	423G ​> ​A		38%
			SNV	426C ​> ​T		37%
			MNV	429_432delGCTTinsAGAA	L144Q	36%
			SNV	439G ​> ​A	V147I	38%
			SNV	441T ​> ​A		38%
			MNV	443_444delATinsGC	N148S	37%
			SNV	447A ​> ​C		36%
			SNV	453A ​> ​T		37%

a AdhE: iron-containing bifunctional aldehyde and alcohol dehydrogenase.

b FliN: Flagellar motor switch.

c Types of secondary mutation: SNV, single nucleotide variation; Ins, insertion; Del, deletion; MNV, two or more SNVs in succession.

d CDS: coding sequence.

e Frequency: sequencing reads containing the mutation as a percentage of all reads that mapped to that location.

The mutation in *adhE* gene found in LL1580, *adhE*^*T585K*^ was previously observed in a strain of *T. saccharolyticum* where the redox-sensing protein Rex had been deleted (Zheng et al., 2018), and was shown to have the effect of eliminating NADH-linked alcohol dehydrogenase activity. Indeed, when we measured ADH activity in LL1580, the NADH-linked activity was eliminated, and we found no difference in the NADPH-ADH activity (Table 4).

**Table 4:** Alcohol dehydrogenase (ADH) specific enzyme activities in wild type T. saccharolyticum with and without ppdk deletion.

	ADH-NADH			ADH-NADPH		
LL1305 (wt)	0.44	(0.28)^a^		0.03	(0.02)	
LL1580 (Δ*ppdk*)	0.02	(0.01)		0.11	(0.14)	

a Numbers indicate average enzyme activity and numbers in parentheses represent the standard deviation (n ​≥ ​3).

The other two mutations found in LL1580 were on a flagellar motor switch gene and on a promotor of region for an RpiR transcriptional regulator. The mutation in the promotor of RpiR is interesting because RipR regulates ribose metabolism and pentose phosphate in *E. coli* and *Staphylococcus aureus* (Sørensen and Hove-Jensen, 1996; Zhu et al., 2011)*.* It will be interesting to study the influence of Δ*ppdk* on ribose metabolism in the future. In LL1686, there are 20 different mutations introduced on the same gene, Tsac_2318. The sequence alignment of Tsac_2318 using BLASTp indicates that it encodes a Uma2 family endonuclease, which is not well studied. Interestingly, Tsac_2318 is upstream of Tsac_2317 encoding for a PTS system transcriptional activator, and the physiologically relevant role of these mutations may be to affect expression of the PTS system, rather than affect the Tsac_2018 gene.

### 3.5 Attempts to delete pyruvate kinase in *T. saccharolyticum*

The results from Δ*ppdk* strains indicates that PPDK is not required for *T. saccharolyticum* metabolism, which leads to the hypothesis that pyruvate kinase is the only essential enzyme involved in glycolysis. To test the hypothesis, we attempted to delete the pyruvate kinase gene in strains LL1305 and LL1328. We designed 4 plasmids to construct deletions of either the entire coding sequence of pyruvate kinase (pJC13) or portions of the coding sequence (pJC15, pJC16, and pJC17). We did not observe any transformants with plasmids pJC13, pJC15 and pJC16. Using pJC17 which creates a 49% truncated pyruvate kinase mutant, we observed a small number of transformants, one of which was isolated and analyzed by whole genome sequencing (LL1609). From the whole genome sequencing, we found that only 34% of the sequencing reads showed the deletion, and the rest still possess the entire pyruvate kinase coding sequence. This appears to result from a nonsense mutation in the counter-selection marker gene, *tdk.* The pyruvate kinase activities in the cell-free extract of LL1609 still shows residual PYK activity (data not shown). We also tried to delete pyruvate kinase gene on top of the *ppdk* deletion in the wild type background (LL1580). Again, we only observed colonies transformed with plasmid pJC17 and we isolated of these colonies for whole genome sequencing (strain LL1610). Similar to strain LL1609, only 50% of the reads in strain LL1610 showed deletion and PYK activity was still present. We also tried to delete the pyruvate kinase gene with a kanamycin positive selection marker and with supplementation of 60 ​mM pyruvate but could not get any correct colonies.

The pyruvate kinase gene is on the same operon downstream of the phosphofructokinase (PFK) gene, which is another essential gene in glycolysis. Typically, both PFK and PYK are key enzymes in the control of glycolysis, and the fact that they are on the same operon suggests coregulation. Based on our failed attempt to delete pyruvate kinase, we believe that it is essential for *T. saccharolyticum*.

## 4 Conclusions

We deleted the *ppdk* gene in wild type and homoethanologen strains of *T. saccharolyticum* and showed that PPDK activity is not essential for growth and is not required for high yield ethanol fermentation.

## CRediT authorship contribution statement

**Jingxuan Cui:** Conceptualization, Investigation, Formal analysis, Writing - original draft. **Marybeth I. Maloney:** Investigation. **Daniel G. Olson:** Conceptualization, Supervision, Writing - review & editing. **Lee R. Lynd:** Conceptualization, Funding acquisition.

## Declaration of competing interest

Lee R. Lynd is a co-founder of the Enchi corporation, which has a financial interest *in Clostridium thermocellum* and *Thermoanaerobacterium saccharolyticum.*
