# Supplementary material for: Conversion of phosphoenolpyruvate to pyruvate in Thermoanaerobacterium saccharolyticum
Source: Metab Eng Commun. 2020 Jan 23;10:e00122. doi: 10.1016/j.mec.2020.e00122 (PMC6997586; doi:10.1016/j.mec.2020.e00122)
Supplement: Multimedia component 1 [file mmc1.docx]

Supplementary information

**Supplementary Table 1. Enzyme activity**

| Strain ID | PYK | PPDK |
| --- | --- | --- |
| LL1305 | 0.42 (0.25) | 0.22 (0.14) |
| LL1580 | 0.49 (0.26) | 0 (0) |
| LL1328 | 0.64 (0.38) | 0.16 (0.03) |
| LL1686 | 0.59 (0.16) | 0 (0) |

**Supplementary Table 2. Fermentation products**

| Substrate Concentration | Strain ID | Consumed Cellobiose  (mM) | Acetate  (mM) | Ethanol  (mM) | Formate  (mM) | Lactate  (mM) |
| --- | --- | --- | --- | --- | --- | --- |
| 5 g/L | LL1305 | 14.65 (0.32) | 12.38 (0.45) | 24.17 (1.50) | 1.11 (0.37) | 8.21 (2.07) |
|  | LL1580 | 14.80 (0.00) | 11.13 (1.38) | 28.83 (2.46) | 1.75 (0.20) | 3.48 (3.00) |
|  | LL1328 | 14.24 (0.38) | 3.15 (0.64) | 42.77 (1.48) | 2.91 (1.67) | 0.00 (0.00) |
|  | LL1686 | 13.39 (0.04) | 0.74 (0.02) | 44.59 (0.92) | 6.02 (0.38) | 0.00 (0.00) |
| 20 g/L | LL1305 | 17.29 (2.77) | 17.94 (2.69) | 36.39 (3.30) | 0.00 (0.00) | 8.98 (3.02) |
|  | LL1580 | 19.86 (2.6) | 16.43 (2.52) | 47.55 (6.70) | 0.00 (0.00) | 12.55 (1.68) |
|  | LL1328 | 61.64 (0.45) | 7.02 (0.71) | 182.92 (15.48) | 8.55 (3.34) | 0.00 (0.00) |
|  | LL1686 | 60.34 (2.13) | 1.65 (1.43) | 173.47 (26.99) | 8.98 (1.79) | 0.00 (0.00) |

**Supplementary Figure 1.** Plasmid design diagram. # is 12, 13, 15, 16, or 17. The *E. coli* replication origin is p15A. Homology sequences were different for each gene of interest, and are all about 500 bp. Selection cassette including Kan promoter, *htk* gene and *tdk* gene was the same as the plasmid backbone pLT26 (Genbank KX272604) (20)
